# The Surface Photogalvanic and Photon Drag Effects in Ag/Pd Metal-Semiconductor Nanocomposite

**DOI:** 10.3390/nano11112827

**Published:** 2021-10-25

**Authors:** Aleksandr S. Saushin, Gennady M. Mikheev, Viatcheslav V. Vanyukov, Yuri P. Svirko

**Affiliations:** 1 Institute of Photonics, University of Eastern Finland, FI-80101 Joensuu, Finland; viatcheslav.vanyukov@uef.fi (V.V.V.); yuri.svirko@uef.fi (Y.P.S.); 2 Institute of Mechanics, Udmurt Federal Research Center of the Russian Academy of Sciences, 426067 Izhevsk, Russia; mikheev@udman.ru

**Keywords:** photon drag effect, surface photogalvanic effect, metal-semiconductor nanocomposite, polarization, photocurrent, mid-IR excitation, nonlinear susceptibility

## Abstract

We performed the investigation of the polarization-sensitive photocurrent generated in silver-palladium metal-semiconductor nanocomposite films under irradiation with nanosecond laser pulses at the wavelength of 2600 nm. It is shown that in both the transverse and the longitudinal configuration, the surface photogalvanic (SPGE) and photon drag effects (PDE) contribute to the observed photocurrent. However, the temporal profile of the transverse photocurrent pulse is monopolar at any polarization and angle of incidence, while the temporal profile of the longitudinal photocurrent pulse depends on the polarization of the excitation beam. Specifically, the irradiation of the film with the *s*-polarized excitation beam produces a monopolar photoresponse, while at *p*-polarized excitation, the photoresponse is bipolar, having a short front and long tail. Obtained experimental results are in agreement with the developed phenomenological theory, which describes transverse and longitudinal photocurrents due to SPGE and PDE in terms of relevant second-order nonlinear susceptibilities and allows us to obtain their dependences on the angle of incidence and polarization of the excitation laser beam. The pronounced dependence of the photocurrent on the angle of incidence and polarization of the excitation beam opens avenues toward the development of polarization- and position-sensitive detectors for industrial and space applications.

## 1. Introduction

Irradiation of the metallic and semiconductor materials with laser pulses can lead to the generation of a photocurrent, which depends on the polarization and incidence angle of the laser beam. This phenomenon can be described in terms of the second-order susceptibility of the medium that can be revealed from the measured photocurrent. The studying of the polarization-sensitive photocurrents is of interest for photonics and optoelectronics, in particular, for THz generation and the development of advanced optoelectronic devices.

On the microscopic level, the polarization-sensitive photocurrent is a manifestation of the sensitivity of the elementary processes of the elastic and non-elastic electron-photon scattering to the photon momentum and to the crystal symmetry [1]. In a centrosymmetric medium, the light scattering cross-section is the same for charge carriers propagating in the opposite directions, i.e., photoexcitation can not lead to the bulk photoresponse. However, in the subsurface layer, the interface lifts the inversion symmetry giving rise to the surface photogalvanic effect (SPGE), which manifests itself as the photocurrent flowing along the semiconductor surface [2]. The transfer of photons momenta to the free carriers is referred to as the photon drag effect (PDE) [3,4], which leads to the photocurrent in the conductive medium. Both SPGE and PDE are second-order nonlinear optical effects that can be observed in non-centrosymmetric and centrosymmetric media [5].

It is worth noting that the amplitude of the SPGE and PDE photocurrent is proportional to the power of the incident light beam, similar to the conventional photovoltaic photodetectors. However, there is an important difference. In conventional semiconductor photodetectors, the electrons and holes generated due to interband transition are separated by the intrinsic electric field at the *p-n* junction. This effect is essentially incoherent, i.e., the photovoltage is determined by the number of photogenerated carriers or the number of absorbed photons. On the contrary, PDE and SPGE are coherent effects. They manifest themselves in the photocurrent, which determined not only the number of incident photons but also their momentum and angular momentum (i.e., the direction of propagation and polarization of the incident light wave).

**Figure 1:**
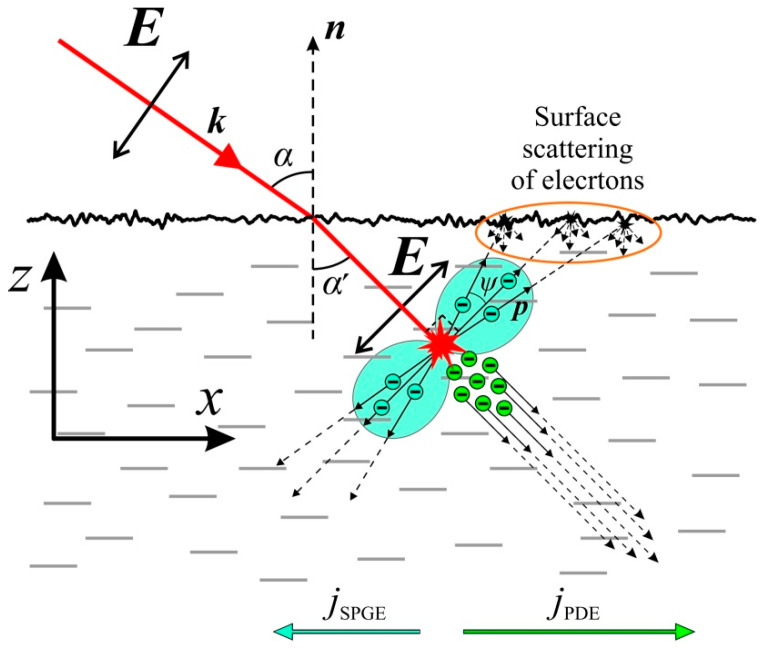
Schematic illustration of SPGE and PDE at the *p*-polarized excitation. The plane of incidence coincides with the (*xz*) plane of the laboratory Cartesian frame. The probability of the photoexcitation of charge is proportional to cos^2^(*ψ*), where *ψ* is the angle between the electric field ***E*** and the electron quasimomentum ***p***. The momenta of photoexcited electrons become unbalanced because the electrons moving toward the surface lose momentum faster than those moving outwards. The PDE photocurrent arises due to free electrons in the sample getting momentum from photons. (Reprinted from [14]).

The microscopic mechanism of the SPGE can be explained by considering photoexcitation of the semiconductor with a *p*-polarized laser beam at oblique incidence (Figure 1). If the semiconductor bandgap is less than the photon energy, the probability of interband transitions will be proportional to (***E***⋅***p***)^2^∝cos^2^*ψ*, where ***E*** is the electric field strength, ***p*** the electron quasimomentum, *ψ* is the angle between ***E*** and ***p*** [6]. Although, for the *p*-polarized beam, the probability of the photoexcitation will be the same for electrons moving toward and outwards the surface, the former will lose their momentum faster because of diffuse scattering on the surface. Such an imbalance will result in a directed flow of electrons along the *x*-axis, i.e., the photocurrent will appear. It is worth noting that this photocurrent vanishes if the excitation beam is *s*-polarized and/or if the reflection of electrons from the surface is specular, i.e., the *x*-component of the electron velocity does not change. The theory of the SPGE has been developed in references [2,7,8]. Experimentally SPGE has been demonstrated in gallium arsenide [9], copper crystal [6], and CuSe films [10,11]. Usually, SPGE is accompanied by the PDE and bulk photogalvanic effect (for example, see references [6,12,13]).

Similar to the SPGE, the PDE manifests itself as a light-induced current generated in solids under irradiation with a laser pulse. However, in contrast to the SPGE, the underlying physical mechanism of the PDE is the photon momentum transfer to the conduction electrons (Figure 1) [3,15]. In semiconductors, the PDE is a consequence of the momentum-selective interband and intraband transitions and different mobility of carriers at the corresponding levels [16,17,18]. The electron drag by photons was first described in reference [19]. The PDE was found in semiconductors (for example, *p*-InSb [20], GaAs [21], tellurium [22,23], Bi [24]), in semiconductor quantum structures GaAs/AlGaAs [25], in metals [6,26], in plasma [27] and in two-dimensional structures of different composition [28,29,30,31], including films of bismuth (semimetal) [32] and metals [29,33], as well as in metal nanowires [34].

The high conductivity of metals makes registration of the PDE and SPGE photocurrents a difficult task because of short-circuiting of the currents in the bulk of the sample [6]. Nevertheless, in a thin film with a surface resistivity of several tens of Ω/□, the photocurrents can be observed by using a broadband oscilloscope [35]. In the thin-film geometry, one can measure longitudinal and transversal photocurrents, which flow along and perpendicular to the incidence plane of the excitation laser beam, respectively.

The silver-palladium (Ag/Pd) nanocomposite films, which are widely used in electronics [36], are one of the materials where both SPGE and PDE can be observed. Being a metal-semiconductor nanocomposite, the Ag/Pd films consist of nanocrystallites of Ag-Pd solid solution, palladium oxide, and amorphous phase, including various oxides [37,38,39]. Conductivity, size, and thickness of the Ag/Pd nanocomposite films may vary in a wide range, while their electronic properties can be controlled in the process the fabrication making these films very suitable for observation of the polarization-sensitive photocurrents. In recent works, we have shown that in the Ag/Pd nanocomposite, one can observe both longitudinal and transverse polarization-sensitive photocurrent in a wide spectral range. We observed the monopolar and bipolar longitudinal photocurrent pulses at *s*- and *p*-polarized excitation beam, respectively, [14] due to the interplay of the PDE and SPGE. However, the contribution of SPGE to the transverse photocurrent was not taken into account, even though the photocurrent has been observed in a wide spectral range of 266–2100 nm [40,41,42]. This was partially because the longitudinal and transverse photocurrents were described separately, i.e., the fact that the nonlinear optical processes manifestations are of the same order was not taken into consideration.

Here, we experimentally study the dependences of longitudinal and transverse photocurrents in the Ag/Pd nanocomposite film on the polarization and angle of incidence of the excitation beam at the wavelength of 2600 nm. We also developed the phenomenological theory of the SPGE and PDE and obtained equations for both longitudinal and transverse photocurrents. It allows us to reveal the values of the nonlinear susceptibilities that determine the polarization-sensitive photoresponse of the Ag/Pd nanocomposite.

## 2. Materials and Methods

**Figure 2:**
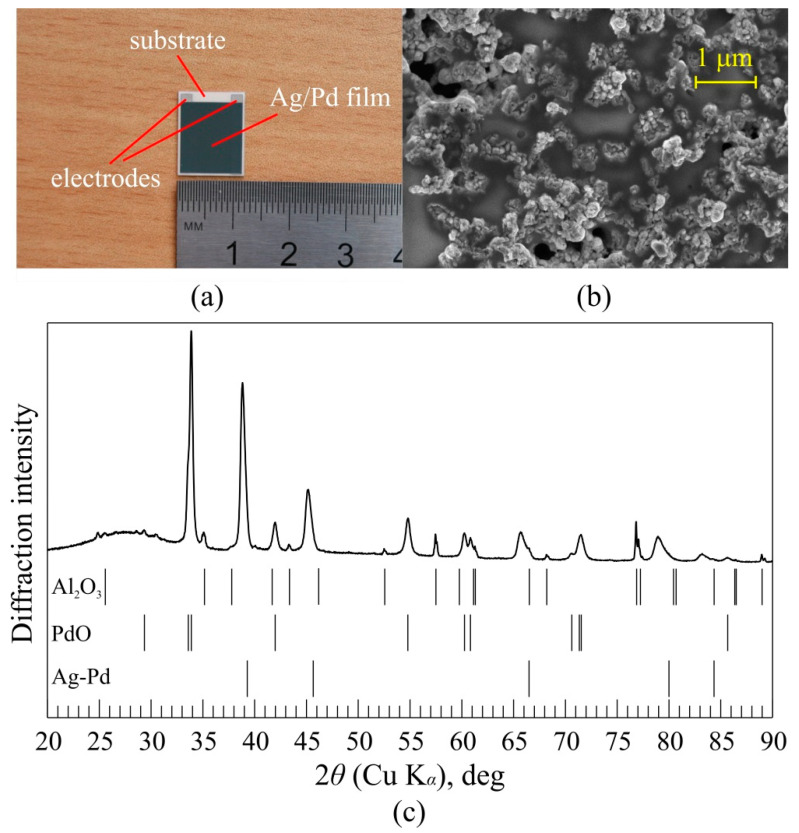
(**a**) The Ag/Pd film with two parallel silver electrodes deposited on the sample’s edges beneath the film; (**b**) scanning electron microscope image of the Ag/Pd film; (**c**) X-ray diffraction patterns of the Ag/Pd film and bar diffraction patterns of detected phases (CuK_α_). Al_2_O_3_ peaks correspond to the diffraction on the dielectric substrate.

We studied the Ag/Pd films fabricated by thick-film technology [36]. In brief, 10 μm thick layer of the paste containing 17.78 wt.% silver oxide, 18.05 wt.% palladium, 44.17 wt.% glass particles, and 20 wt.% organic binder was applied on a ceramic substrate and annealed at the temperature of 833 K. To enable electrical measurements, two parallel silver electrodes were deposited on the sample’s edges beneath the film (see Figure 2a), which had an area of 10 × 12 mm^2^. Our measurement revealed that the film possesses *p*-type conductivity at the carrier concentration of 9.2 × 10^20^ cm^−3^, mobility of 10^−1^ cm^2^/(V∙s), and resistivity of 6.6 × 10^−2^ Ω∙cm.

Figure 2b shows the scanning electron microscope image of the film surface. The X-ray diffraction measurements (see Figure 2c) revealed that the fabricated Ag/Pd films include Ag-Pd solid solution and PdO nanocrystallites having a lateral dimension of 35 nm.

In the experiment, we employed nanosecond pulses of the optical parametric generator tunable in the wavelength range of 1350–5000 nm having the pulse duration of τ = 7 ns. Since both SPGE and PDE are expected to manifest in the IR range [14], the light-induced current was studied at the irradiation of the Ag/Pd films at the wavelength of 2600 nm.

**Figure 3:**
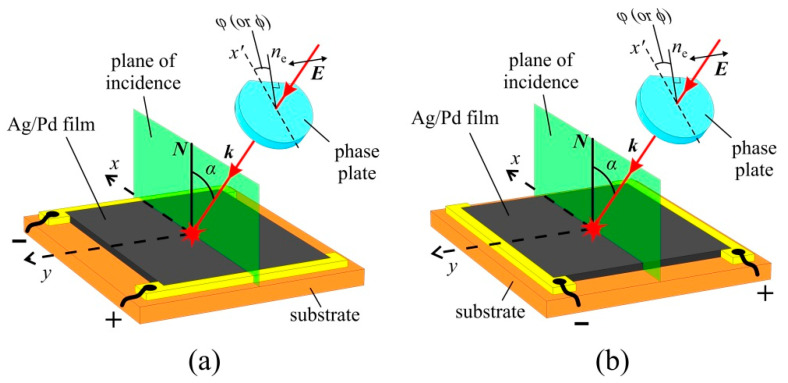
Sketch of the experiments for registration of the (**a**) longitudinal and (**b**) transverse photocurrents in the Ag/Pd nanocomposite films. The electrodes are oriented (**a**) perpendicular and (**b**) parallel to the plane of the incidence, which coincides with the *xz* plane of the laboratory Cartesian frame. ***E*** is the electric field vector of the beam before a wave plate, which is perpendicular to the plane of incidence, ***k*** is wave vector, *n*_e_ is the slow axis of the wave plate, ***N*** is a unit vector of normal to the film surface, *α* is the angle of incidence, *x*’ is an axis in the plane of incidence and perpendicular to the laser beam. The polarization of the exciting radiation was determined by the angle *φ* or *ϕ* of the orientation of the quarter-wave plate or half-wave plate, respectively.

The photovoltage generated between electrodes was measured as a function of the incidence angle α and polarization state of the excitation beam (Figure 3). The latter was determined by the orientation of the slow axes of the half- or quarter-wave plates. By rotating the phase plate around the beam, one can change the polarization plane azimuth or the degree of circular polarization.

The Ag/Pd film was placed on a special goniometric holder, which permits rotating the sample around the surface normal and changing the incidence angle (the place of irradiation was not changed). The diameter of the laser beam was 2.6 mm, and the electrodes were not irradiated at *α* < 70°.

In the experiment, we measured photovoltages $U_{x}$ and $U_{y}$ generated between electrodes when the film is irradiated by the laser pulse in the longitudinal (Figure 3a) and transverse (Figure 3b) configurations, respectively. The electrodes were connected to a LeCroy WaveSurfer 42Xs oscilloscope with 400 MHz bandwidth at input resistance of *R*_in_ = 50 Ω. The measured signals were averaged over 100 laser pulses. Since the photocurrent was a linear function of the laser pulse energy [42,43], it is convenient to introduce the longitudinal (Figure 3a) and transverse (Figure 3b) light-to-current conversion efficiencies as:

(1) $$\eta_{x}=\frac{U_{x}}{R_{in}W_{in}},\;\eta_{y}=\frac{U_{y}}{R_{in}W_{in}}\;,$$

where *W_in_* is the excitation pulse energy measured by the pyroelectric energy detector QE25HR-H-MB-D0 (Gentec).

## 3. Phenomenological Theory of the Light-Induced Current in the Ag/Pd Nanocomposite

### 3.1. SPGE

The Ag/Pd film comprises metal and semiconductor nanocrystallites embedded into an amorphous glassy matrix. Since the size of the metal and semiconductor nanocrystallites is much smaller than the excitation wavelength and the laser spot size, we can use an effective medium approach to describe the photoresponse of the film. Specifically, we consider the Ag/Pd nanocomposite as an isotropic medium having complex dielectric constant and nonlinear susceptibility, which are determined by the concentration and size of the nanocrystallites. Since Ag-Pd and PdO nanocrystallites constituting the film are centrosymmetric, the bulk photogalvanic effect in the Ag/Pd nanocomposite is forbidden. According to reference [2], the SPGE surface current density is described by the following constitutive equation:

(2) $$j_{i}^{\left(\mathrm{SPGE}\right)}=\theta_{ii^{\prime }}G_{i^{\prime }klm}N_{m}E_{k}^{\left(t\right)}E_{l}^{\left(t\right)*},\;$$

where subscripts label axes of the laboratory Cartesian frame, ***N*** is a unit vector along the film normal. Tensor $\theta_{ij}=\delta_{ij}-N_{i}N_{j}$ describes the current along the surface, ***E***^(***t***)^ is the vector of the complex amplitude of the electric field strength inside the medium. The reality of the SPGE current implies that second-order susceptibility tensor $G_{ijlm}$ satisfies the symmetry relation $G_{ijlm}=G_{iljm}^{*}$.

In the isotropic medium, only three components of the fourth rank tensor $G_{ijlm}$ are independent. In the laboratory Cartesian frame having *z*-axes along ***N*** and *xz* plane coinciding with the incidence plane (see Figure 3), the longitudinal ($j_{x}^{\left(\mathrm{SPGE}\right)}$) and transversal ($j_{y}^{\left(\mathrm{SPGE}\right)}$) components of the SPGE surface current density are determined by the only component $G_{xxzz}$ of the nonlinear susceptibility tensor [44]:

(3) $$j_{x}^{\left(\mathrm{SPGE}\right)}=2\;Re\;\left\{G_{xxzz}E_{x}^{\left(t\right)}E_{z}^{\left(t\right)*}\right\},\;j_{y}^{\left(\mathrm{SPGE}\right)}=2\;Re\;\left\{G_{xxzz}E_{y}^{\left(t\right)}E_{z}^{\left(t\right)*}\right\}.\;$$

Photovoltages $U_{x}^{\left(\mathrm{SPGE}\right)}$ and $U_{y}^{\left(\mathrm{SPGE}\right)}$ generated between electrodes due to the SPGE currents flowing along (Figure 3a) and perpendicular (Figure 3b) incidence plane, respectively, are:

(4) $$U_{x,y}^{\left(\mathrm{SPGE}\right)}=j_{x,y}^{\left(\mathrm{SPGE}\right)}\frac{\rho \;S}{LD},\;$$

where ρ, S and *D* are the resistivity, irradiated area and thickness of the film, respectively, *L* is the length of the electrodes. By taking into account that the irradiated film area is $S=S_{0}/\cos\alpha$, where $S_{0}$ is the laser beam cross-section, we arrive at the following equations for the SPGE conversion efficiencies in the longitudinal and transversal configurations:

(5) $$\eta_{x}^{\left(\mathrm{SPGE}\right)}=A_{\mathrm{SPGE}}\frac{{\left|E_{p}\right|}^{2}}{{\left|E\right|}^{2}},$$

(6) $$\eta_{y}^{\left(\mathrm{SPGE}\right)}=Re\left\{B_{\mathrm{SPGE}}\frac{E_{p}^{*}E_{s}}{{\left|E\right|}^{2}}\right\}.$$

Here $E_{p}$ and $E_{s}$ are amplitudes of the *p*- and *s*-polarized components of the incident light wave, respectively, (see Equations (A14)–(A19) in Appendix A), ${\left|E\right|}^{2}={\left|E_{s}\right|}^{2}+{\left|E_{p}\right|}^{2}$, and

(7) $$A_{\mathrm{SPGE}}=\frac{\rho Z_{0}}{2\tau LDR_{in}}\frac{8m^{2}\sin\alpha \cos\alpha }{{\left|n\cos\alpha +\cos\alpha_{t}\right|}^{2}}Re\left\{\frac{G_{xxzz}}{n^{*}}\cos\alpha_{t}\right\},$$

(8) $$B_{\mathrm{SPGE}}=\frac{\rho Z_{0}}{2\tau LDR_{in}}\frac{8m\;G_{xxzz}\sin\alpha \cos\alpha }{\left(n^{*}\cos\alpha +\cos\alpha_{t}^{*}\right)(\cos\alpha +n\cos\alpha_{t})n^{*}},$$

where $Z_{0}=\sqrt{\mu_{0}/\varepsilon_{0}}=377\;\Omega$ is the vacuum impedance, and $\sin\alpha_{t}=\sin\alpha /n$, $m={\left({\left|\sin\alpha_{t}\right|}^{2}+{\left|\cos\alpha_{t}\right|}^{2}\right)}^{-1/2}$.

### 3.2. PDE

The PDE is the second-order nonlinear optical phenomenon. The PDE current density can be presented by the following constitutive equation (see, for example, [22]):

(9) ji(PDE)=∑j,k,m=x,y,zIm{ΓijlmEj(t)*∇mEl(t)},

where $\Gamma_{ijlm}$ is the tensor of the nonlinear susceptibility. In the isotropic film, only three components of this material tensor are independent, and Equation (9) can be reduced down to:

(10) $$j_{i}^{\left(\mathrm{PDE}\right)}=\sum_{j=x,y,z}^{}Im\left\{\Gamma_{xxyy}\;E_{i}^{\left(t\right)*}\nabla_{j}\;E_{j}^{\left(t\right)}+\Gamma_{xyxy}E_{j}^{\left(t\right)*}\nabla_{j}\;E_{i}^{\left(t\right)}+\Gamma_{xyyx}E_{j}^{\left(t\right)*}\nabla_{i}\;E_{j}^{\left(t\right)}\right\}.$$

The current depends on the relationship between electron mean free path *Λ* and absorption length *d*. If *d* is less or of the same order as *Λ*, we can consider only electron movement in the lateral plane, i.e., in Equation (9), summation should include only *x* and *y* axes [45]. In reference [37], it was shown that the Ag/Pd films consist of PdO, and the Ag-Pd solid solution has 59% of the total Ag content. In silver, the electron mean free path *Λ* = 57 nm [46], while the light penetration depth *d* = 12 nm [47]. Therefore, one may expect that in the Ag/Pd films, *d* is less or the same order as *Λ*. Correspondingly, in the laboratory Cartesian frame with *z*-axis along film normal and *xz* plane coinciding with incident one, the components of the longitudinal ($j_{x}^{\left(\mathrm{PDE}\right)}$) and transversal ($j_{y}^{\left(\mathrm{PDE}\right)}$) PDE current density can be presented in the following form:

(11) $$j_{x}^{\left(\mathrm{PDE}\right)}=\frac{\omega }{c}Re\;\left\{\left(\Gamma_{xxyy}+\Gamma_{xyxy}+\Gamma_{xyyx}\right){\left|E_{x}^{\left(t\right)}\right|}^{2}+\Gamma_{xyyx}{\left|E_{y}^{\left(t\right)}\right|}^{2}\right\}\sin\alpha ,$$

(12) $$j_{y}^{\left(\mathrm{PDE}\right)}=\frac{\omega }{c}Re\;\left\{\Gamma_{xxyy}E_{x}^{\left(t\right)}E_{y}^{\left(t\right)*}+\Gamma_{xyxy}E_{x}^{\left(t\right)*}E_{y}^{\left(t\right)}\right\}\sin\alpha .\;$$

The voltages $U_{x}^{\left(\mathrm{PDE}\right)}$ and $U_{y\;}^{\left(\mathrm{PDE}\right)}$ generated between electrodes due to PDE currents flowing along (Figure 3a) and perpendicular (Figure 3b) incidence plane, respectively, are:

(13) $$U_{x,y}^{\left(\mathrm{PDE}\right)}=j_{x,y}^{\left(\mathrm{PDE}\right)}\frac{\rho Sd}{LD}.$$

By using Equation (10) and Equations (A14)–(A19), we arrive at the following equations for the PDE conversion efficiencies in the longitudinal and transversal configurations:

(14) $$\eta_{x}^{\left(\mathrm{PDE}\right)}=\left[A_{\mathrm{PDE}}^{\left(1\right)}\frac{{\left|E_{p}\right|}^{2}}{{\left|E\right|}^{2}}+A_{\mathrm{PDE}}^{\left(2\right)}\frac{{\left|E_{s}\right|}^{2}}{{\left|E\right|}^{2}}\right],$$

(15) $$\eta_{y}^{\left(\mathrm{PDE}\right)}=Re\left\{B_{\mathrm{PDE}}\frac{E_{p}^{*}E_{s}}{{\left|E\right|}^{2}}\right\},$$

where

(16) $$A_{\mathrm{PDE}}^{\left(1\right)}=\frac{\rho dZ_{0}}{2\tau LDR_{in}}\frac{\omega }{c}\frac{4m^{2}\left|\cos^{2}\alpha_{t}\right|\sin\alpha \cos\alpha }{{\left|n\cos\alpha +\cos\alpha_{t}\right|}^{2}}Re\left\{\Gamma_{xxyy}+\Gamma_{xyxy}+\Gamma_{xyyx}\right\},$$

(17) $$A_{\mathrm{PDE}}^{\left(2\right)}=\frac{\rho dZ_{0}}{2\tau LDR_{in}}\frac{\omega }{c}\frac{4\sin\alpha \cos\alpha }{{\left|n\cos\alpha_{t}+\cos\alpha \right|}^{2}}Re\left\{\Gamma_{xyyx}\right\},$$

(18) $$B_{\mathrm{PDE}}=\frac{\rho dZ_{0}}{2\tau LDR_{in}}\frac{\omega }{c}\frac{4m\sin\alpha \cos\alpha \cos^{*}\alpha_{t}}{\left(n^{*}cos\;\alpha +\cos\alpha_{t}^{*}\right)(\cos\alpha +n\cos\alpha_{t})}\left(\Gamma_{xxyy}^{*}+\Gamma_{xyxy}\right).$$

### 3.3. Polarization-Sensitive Photoresponse

In this paper, we consider the control of the polarization state of the excitation wave by rotating half- and quarter-wave plates.

When the beam is linearly polarized, its polarization azimuth is determined by the rotation of the half-wave (HW) plate slow axis by ϕ (see Figure 3). By using Equations (A14)–(A19), we arrive at the following equations for the conversion efficiencies in the longitudinal and transversal configurations:

(19) $$\eta_{x}^{HW}=\left(A_{\mathrm{SPGE}}+A_{\mathrm{PDE}}^{\left(1\right)}\right)\sin^{2}2\phi +A_{\mathrm{PDE}}^{\left(2\right)}\cos^{2}2\phi ,$$

(20) $$\eta_{y}^{HW}=Re\left\{B_{\mathrm{SPGE}}+B_{\mathrm{PDE}}\right\}\sin2\phi \cos2\phi .\;$$

After the quarter-wave (QW) plate has a slow axis rotated by φ, the excitation beam is elliptically polarized, and conversion efficiencies are

(21) $$\eta_{x}^{QW}=\frac{1}{2}\;\left[\left(A_{\mathrm{SPGE}}+A_{\mathrm{PDE}}^{\left(1\right)}\right)\sin^{2}2\varphi +A_{\mathrm{PDE}}^{\left(2\right)}\left(1+\cos^{2}2\varphi \right)\right],$$

(22) $$\eta_{y}^{QW}=\frac{1}{2}\left[Re\left\{B_{\mathrm{SPGE}}+B_{\mathrm{PDE}}\right\}\sin2\varphi \cos2\varphi -Im\left\{B_{\mathrm{SPGE}}+B_{\mathrm{PDE}}\right\}\sin2\varphi \right].$$

The Equations (19)–(22) allow one to describe the polarization-sensitive photocurrents in the Ag/Pd nanocomposite films.

## 4. Results and Discussion

**Figure 4:**
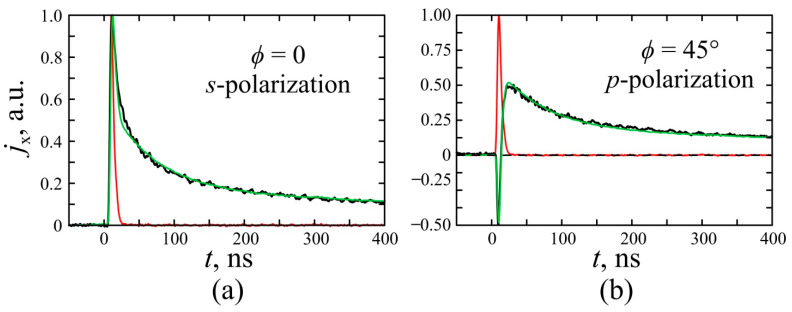
Oscillograms of longitudinal photocurrent pulses produced by a laser beam at different half-wave plate orientations: (**a**) *ϕ* = 0 and (**b**) *ϕ* = 45°. The red lines denote the exciting laser pulse while the black lines show the measured photocurrent pulse, and the green lines are the fitting by Equation (23).

Figure 4 shows the temporal profiles of the photoresponse pulses for *p*- and *s*-polarization at the excitation wavelength of 2600 nm and the incident angle of α=45°. One can see that at *p*-polarized excitation, photocurrent pulses are bipolar and consist of short-front and long-tail parts, while for the *s*-polarized excitation beam, the photoresponse pulse is monopolar. We have recently demonstrated [14] that the bipolar photocurrent pulses are a consequence of the interplay of counter-flowing SPGE and PDE photocurrents generated in the Ag/Pd film. Since the SPGE is forbidden at *s*-polarized excitation, the photoresponse at *s*-polarization can be attributed to PDE. There is a common belief that PDE is the phenomenon with sub-picosecond relaxation time (see, for example [48]), however in our experiments, the decay time of the photoresponse is as long as 1000 ns, i.e., it unlikely originate from the nearly instantaneous PDE effect. The tail part of bipolar pulses has the same decay time and polarity independently of polarization, i.e., it is not due to SPGE as well.

We suggest that such a long tail, which have also been observed at the visual and UV excitation pulse [14,42,49], may originate from the composition of the Ag/Pd film, which consists of metal (Ag-Pd solid solution), semiconductor (PdO), and dielectric (glass) nanoparticles. Since the work function of PdO [50,51] is significantly higher than that of palladium [50] and silver [52], there exists a network of the nanosized Ag/PdO and Pd/PdO Schottky barriers for electrons flowing from the metal to the semiconductor.

When a conductive electron in a metal nanoparticle gains momentum after absorption of a photon, its energy may be enough to overcome the potential barrier at the semiconductor-metal junction, i.e., the electrons can pass semiconductor particles and arrive at the metal nanoparticle surrounded by semiconductor and dielectric. If the electrons lose their energy, they will be locked in the metal nanoparticle; correspondingly, the initial metal particle acquires a positive charge, and the particle that caught the electrons gets a negative charge. This will produce the electric field directed along the electron velocity, i.e., the voltage between electrodes will be created. The leakage of the electrons through the metal nanoparticle boundary will manifest itself as slow decay of the PDE voltage measured by the oscilloscope. It is worth noting that the tail of the photoresponse pulse associated with PDE may significantly exceed the duration of the excitation pulse. However, one may suggest that the potential barrier at the metal-semiconductor interface may be suppressed or even vanish if the metal nanoparticle accumulated a sufficient number of electrons. In such a case, the electrons can leave the trap as soon as their number exceeds a threshold, which is determined by the particle size. As a result, the amplitude of the long-lasting tail of the PDE photoresponse will show a weak dependence on the excitation beam polarization.

The described above mechanism of the photoresponse pulse elongation does not work for the SPGE. This is because the SPGE originates from the asymmetry of the scattering of the photoelectrons propagating toward and away from the surface (see Figure 1). When metal nanoparticles are sandwiched between two semiconductor nanoparticles, the metal nanoparticle will catch an equal number of photoelectrons from each metal-semiconductor junction. Since photoelectrons generated in each semiconductor nanoparticle propagate in opposite directions, the resulting electric field generated at such a double Schottky barrier will be zero. That is, the SPGE photoresponse will have no long-lasting tail, and the photocurrent pulse will reproduce the shape of the excitation laser pulse.

To summarize, the rise of the photoresponse is determined by the front of the nanosecond excitation pulse, while the tail of the photoresponse is governed by a slow relaxation of the PDE photocurrent due to Schottky traps. This allows us to describe the temporal profile of the longitudinal photocurrent pulse by the following equation (Figure 4):

(23) $$\eta_{x}^{HW}\left(\phi ,\alpha ,t\right)=\frac{Z_{0}}{2}\overset{\infty }{\underset{0}{\int }}I\left(t-\tau \right)\left\{\left(A_{\mathrm{PDE}}^{\left(1\right)}+A_{\mathrm{PDE}}^{\left(2\right)}\right)\cos^{2}2\phi \left[\left(1-a_{\mathrm{Ag}}-a_{\mathrm{Pd}}\right)\exp\left(-\frac{\tau }{T_{\mathrm{PDE}}}\right)+a_{\mathrm{Ag}}\cdot \exp\left(-\frac{\tau }{T_{\mathrm{Ag}}}\right)+\;a_{\mathrm{Pd}}\cdot \exp\left(-\frac{\tau }{T_{\mathrm{Pd}}}\right)\right]-A_{\mathrm{SPGE}}\sin^{2}2\phi \exp\left(-\frac{\tau }{T_{\mathrm{SPGE}}}\right)\right\}d\tau ,\;$$

where I(t) describes the shape of the excitation pulse, $T_{\mathrm{PDE}}$ and $T_{\mathrm{SPGE}}$ are PDE and SPGE relaxation times; correspondingly, $a_{\mathrm{Ag}}$ and $a_{\mathrm{Pd}}$ correspond to the electrons locked by Ag/PdO and Pd/PdO traps, respectively, and *T*_Ag_ and *T*_Pd_ are relaxation times of Ag/PdO and Pd/PdO traps, consistently. Green lines in Figure 4 show an approximation of pulses temporal profile with Equation (23). One can see that experimental data are in agreement with calculations.

**Figure 5:**
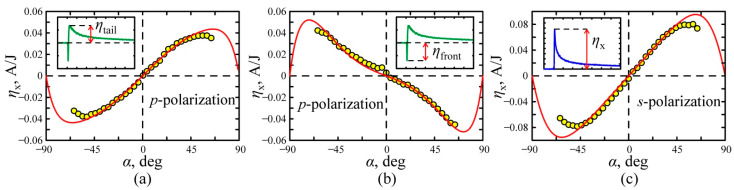
The dependence of the longitudinal light-to-current conversion efficiency on the incidence angle α for amplitude of the (**a**) tail part and (**b**) front part of the photocurrent pulses at the *p*-polarized excitation beam, (**c**) photocurrent pulse at the *s*-polarized excitation beam. The solid lines are approximations with parameters in Table 1. Insets to (**a**–**c**) show temporal profiles of the photocurrent pulse.

Figure 5 shows the dependences of the conversion efficiency on the angle of incidence for the *p*- and *s*-polarized excitation laser beam. Since at *p*-polarized excitation the photoresponse is bipolar, the incidence angle dependences for the long-tail and short-front parts of the photoresponse are shown separately in Figure 5a,b. For the *s*-polarized excitation beam, the photoresponse pulse is monopolar, and its angle dependence is shown in Figure 5c. It is seen that all dependences presented in Figure 5 are odd functions, i.e., $\eta_{x}\;\left(\alpha \right)=-\eta_{x}\;\left(-\alpha \right)$, which is characteristic for PDE and SPGE. To describe the dependences shown in Figure 5, the nonlinear parameters shown in Table 1 were calculated using Equations (7),(19) and (23). One can see that the experimental data (circles) are in suitable agreement with the results of fitting based on the nonlinear parameters of the film shown in Table 1. It is worth noting that since at the normal incidence, the photocurrent is zero irrespectively on the position of the irradiated area on the Ag/Pd film surface, the thermoelectric [53], Dember [54], and the photovoltaic [55] effects do not contribute to the photocurrent.

**Figure 6:**
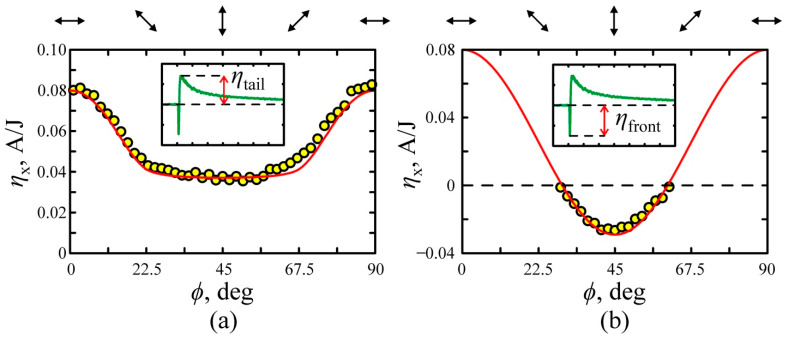
The dependences of the longitudinal conversion efficiency on the half-wave plate orientation *ϕ* for the (**a**) tail and (**b**) front parts of the photocurrent pulses. The circles represent the experimental data, while red lines correspond to approximation by Equations (23) and (24) with parameters given in Table 1. Arrows at the top of (**a**,**b**) show orientation of the polarization azimuth of the excitation beam. Insets to (**a**,**b**) show temporal profiles of the photocurrent pulse.

Figure 6a,b shows the dependences of the longitudinal photoresponse on the half-wave plate orientation ϕ at α=45°. One can see that the tail part of photoresponse is maximal at *s*-polarization and minimal at *p*-polarization. The circles represent experimental results, while red solid lines show results of fitting at parameters shown in Table 1, where the amplitude $\eta_{tail}$ of the tail part of the bipolar photoresponse pulse is found from Equation (23).

One can observe from Figure 6b that the photoresponse pulse is bipolar at 25<ϕ<55°. The negative front part appears if the amplitude of SPGE pulse exceeds the amplitude of PDE photocurrent, it can be described using Equations (7) and (19) in the following form:

(24) $$\eta_{neg}\left(\phi ,\;\alpha \right)=(A_{\mathrm{PDE}}^{\left(1\right)}-A_{\mathrm{SPGE}})\sin^{2}2\phi +A_{\mathrm{PDE}}^{\left(2\right)}\cos^{2}2\phi .$$

**Figure 7:**
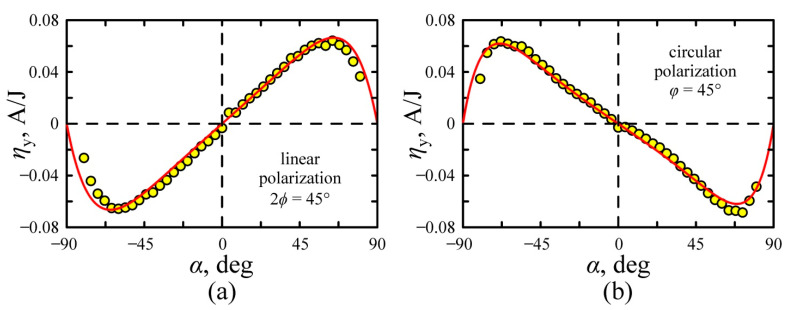
The transverse light-to-current conversion efficiency as a function of incidence angle α: (**a**) linear polarized excitation beam at *ϕ* = 22.5° and (**b**) circular polarized excitation beam (*φ* = 45°). The solid lines are approximations at parameters in Table 1 with Equations (25) and (26).

Numerous experiments have shown that the transverse photocurrent pulses are monopolar at any polarization and angle of incidence. Figure 7 shows the dependences of conversion efficiency for transverse photocurrent on the angle of incidence for linear and circular polarization. It can be seen that the conversion efficiency is an odd function of the incidence angle, i.e., $\eta_{y}\;\left(\varphi ,\;\alpha \right)=-\eta_{y}\;\left(\varphi ,-\alpha \right)$ and there is no photocurrent at α = 0. The angle dependences of transverse photocurrent are well approximated by the following equations:

(25) $$\eta_{y}\left(\alpha \right)=Re\left\{B_{\mathrm{SPGE}}\left(\alpha \right)-B_{\mathrm{PDE}}\left(\alpha \right)\right\},\;\mathrm{for}\;\mathrm{linear}\;\mathrm{polarization},\;$$

(26) $$\eta_{y}\left(\alpha \right)=-Im\left\{B_{\mathrm{SPGE}}\left(\alpha \right)-B_{\mathrm{PDE}}\left(\alpha \right)\right\},\;\mathrm{for}\;\mathrm{circular}\;\mathrm{polarization},\;$$

One can observe from Figure 7 that Equations (25) and (26) with parameters given in Table 1 well describe the experimental results. 

**Figure 8:**
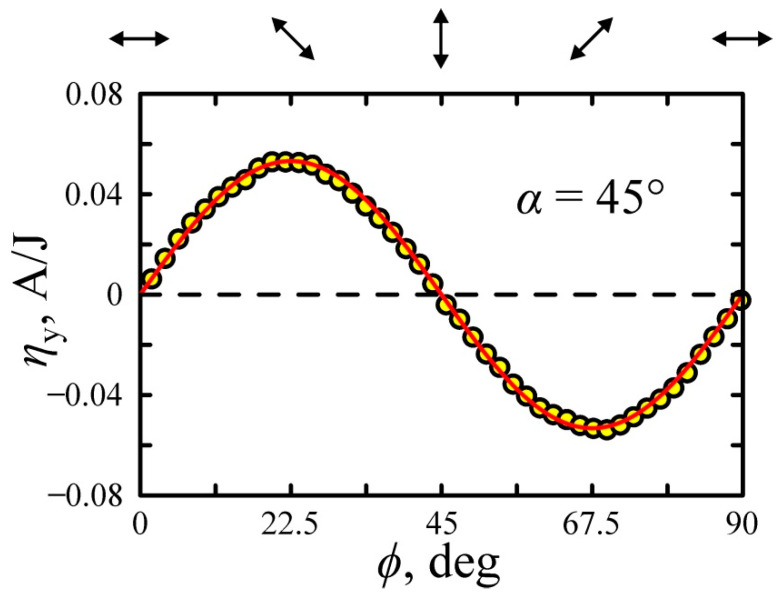
Dependences of the transverse conversion efficiency on the half-wave plate rotation angle *ϕ* at α = 45°. The solid line shows fitting by Equation (27) at parameters in Table 1. Arrows at the top of the figure show orientation of the polarization azimuth of the excitation beam.

Figure 8 shows the dependence of transverse conversion efficiency on the rotation angle of the half-wave plate at α = 45°. One can see that the conversion efficiency is maximum at *ϕ* = 22.5° and 67.5° and is an odd function of *ϕ*, i.e., $\eta_{y}\;\left(\phi \right)=-\eta_{y}\;\left(-\phi \right)$ and $\eta_{y}\;\left(\phi \right)=0$ for the *p*- and *s*-polarized excitation beams. It is worth noting that the experimental data in Figure 8 are well described by the following equation using material parameters listed in Table 1:

(27) $$\eta_{y}\left(\phi \right)=Re\left\{B_{\mathrm{SPGE}}-B_{\mathrm{PDE}}\right\}\sin2\phi \cos2\phi .$$

**Figure 9:**
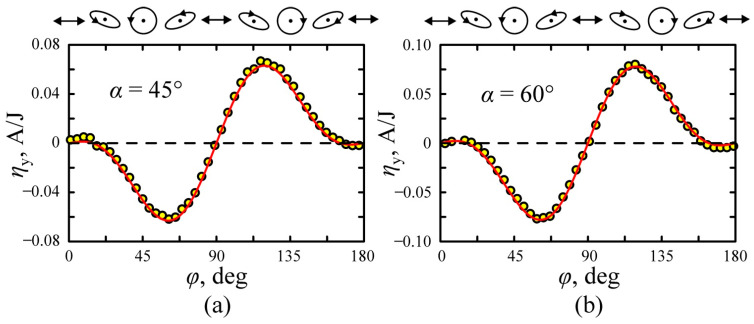
Dependences of the transversal conversion efficiency on quarter-wave plate orientation *φ* at (**a**) α = 45° and (**b**) α = 60°. The solid lines show fitting by Equation (28) at parameters in Table 1. Polarization ellipses as seen by the observer looking at the excitation beam source are shown at the top.

Figure 9a,b shows the dependence of transverse photocurrent on the angle *φ* between slow axis of a quarter-wave plate and plane of incidence at α=45° and α=60°, respectively. Experimental data are well described by the equation

(28) $$\eta_{y}\left(\varphi \right)=\frac{1}{2}\left[Re\left\{B_{\mathrm{SPGE}}-B_{\mathrm{PDE}}\right\}\sin2\varphi \cos2\varphi -Im\left\{B_{\mathrm{SPGE}}-B_{\mathrm{PDE}}\right\}\sin2\varphi \right],$$

using parameters presented in Table 1. It is worth noting that the direction of the photocurrent depends on the direction of electric field vector rotation (helicity) of the laser pumping. The conversion efficiency for the circularly polarized excitation beams $\eta_{y}\left(\varphi =\pm 45{}^{\circ}\right)=Im\left\{B_{\mathrm{SPGE}}-B_{PDE}\right\}$ represents the circular photocurrent, which flows in opposite directions for the left- and right-circular polarized waves.

Thus all experimental data are well described in terms of PDE and SPGE theories at the same nonlinear susceptibilities.

Since the polarization-sensitive photocurrent strongly depends on polarization state and wave vector of exciting light, the research is of interest for optoelectronics, especially for polarization and angle sensors development. In particular, we showed that the direction in which the transverse photocurrent flows depends on whether the electric field vector in the incident waves rotates clockwise or counterclockwise. This experimental finding allows us to distinguish whether the excitation pulse is right- or left-circular polarized by measuring the polarity of the photovoltage generated between electrodes. From the application perspective, this phenomenon can be employed to analyze the helicity of the laser beam.

The results obtained show that the irradiation of Ag/Pd nanocomposite films with nanosecond laser pulses leads to the generation of the photocurrent due to PDE and SPGE. One may expect that irradiation of the film with femtosecond pulses will result in excitation of the ultrafast currents, which will manifest themselves in the THz emission. The THz emission due to the PDE has been extensively studied in graphene, dichalcogenides, and other 2D materials both experimentally and theoretically [56,57,58,59]. However, to the best of our knowledge, the THz emission due to SPGE has not been discussed yet. Since polarization sensitive SPGE and PDE currents in the Ag/Pd film are comparable in amplitude, their interplay may enable all-optical control of the propagation direction and polarization of the THz wave.

## 5. Conclusions

The dependences of photocurrents on polarization and angle of incidence in the Ag/Pd nanocomposite at 2600 nm nanosecond excitation were studied. It is shown that at *p*-polarized excitation, the pulses of longitudinal photocurrent are bipolar and consist of short-front and long-tail parts. The amplitude dependences of short and long parts of the pulse on polarization and angle of incidence were found. The front short part gradually disappears as polarization approaches the *s*-state. At *s*-polarized excitation, the photocurrent pulses are monopolar at any incidence angle. It is shown that pulses of transverse photocurrent are monopolar at any polarization and angle of incidence. The polarity of photocurrent pulses reverses for any polarization of the exciting beam when the sign of the angle of incidence changes.

We described experimental dependences using the phenomenological theory of the PDE and SPGE that presents photocurrent in terms of the nonlinear optical susceptibilities of an isotropic metal-semiconductor nanocomposite. The developed theory allows one to obtain the dependence of the photocurrent on the angle of incidence and polarization of the excitation beam that is in suitable agreement with the experimental data. The results of the performed experiments and the developed theory made it possible to find the nonlinear optical parameters characterizing the Ag/Pd films. The obtained results are promising for future optoelectronics and, particularly, for the development of polarization-sensitive photo.

## Figures and Tables

**Table 1:** The fitting parameters for the experimental data.

*G_xxzz_*, 10^−14^, m/(ΩV)	*Γ_xxyy_*, 10^−14^, m/(ΩV)	*Γ_xyxy_*, 10^−14^, m/(ΩV)	*Γ_xyyx_*, 10^−14^, m/(ΩV)		*n*
4.2 + 3.3i	11.65 − 7.7i	11.65 + 6.4i	164.1 + 76.9i		1.44 − 0.29i
*T*_SPGE_, ns	*T*_PDE_, ns	*T*_Pd_, ns	*T*_Ag_, ns	$a_{\mathrm{Pd}}$	$a_{\mathrm{Ag}}$
1	1.5	65	800	0.269	0.146

**Figure A1:**
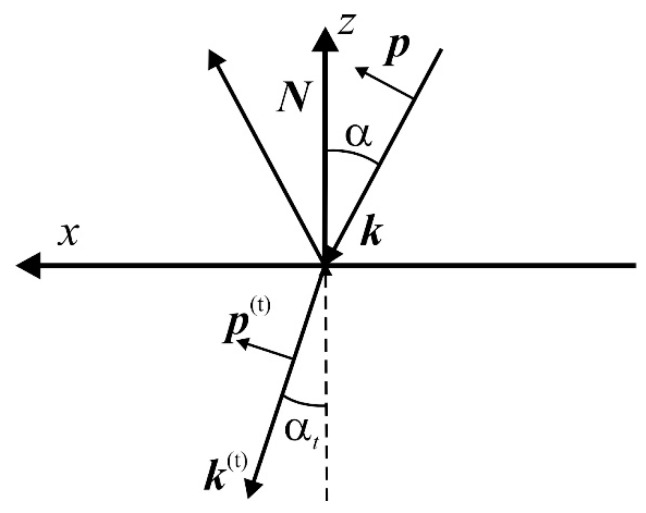
Incidence of the light wave on the interface between isotropic medium and vacuum, α is the angle of the incidence. The electric field of the transversal light wave is perpendicular to the wave vector $K=\frac{\omega }{c}k$, where k is the unit vector along the propagation direction. We introduce *p*- and *s*-polarizations of the incident and transmitted wave. The *s*-polarization is oriented perpendicular to the plane of incidence *xz*.

## Data Availability

The data presented in this study are available on request from the corresponding author.

## Appendix A

If the light obliquely hits the isotropic surface at α (Figure A1), the wave vector of the incident light wave is $K=\frac{\omega }{c}k$, where k={sinα, 0,−cosα} is a unit vector. At any polarization, the incident light can be presented as a combination of *p*- and *s*-polarized waves. Unit vector along *s*-polarization is perpendicular to the wave vector k and to the normal to medium surface N={0, 0, 1}:

(A1) $$s=\frac{\left[k\times N\right]}{\left|\left[k\times N\right]\right|}=\left\{0,-1,\;0\right\}.$$

That is, the amplitude of the *s*-polarized component of the incident wave is

(A2) $$E_{s}=\left(Es\right)=-E_{y}.$$

The unit vector of the *p*-polarization is perpendicular to that of *s*-polarization- and can be expressed in the form:

(A3) $$p=\frac{\left[s\times k\right]}{\left|\left[s\times k\right]\right|}=\frac{N-k\left(kN\right)}{\sqrt{1-{\left(kN\right)}^{2}}}=\left\{\cos\alpha ,0,\sin\alpha \right\}.$$

Correspondingly, the amplitude of the *p*-polarized component of the incident wave:

(A4) $$E_{p}=\left(Ep\right)=E_{x}\cos\alpha +E_{z}\sin\alpha .$$

The vector of the electric field of the incident wave can be presented in the following form:

(A5) $$E=sE_{s}+pE_{p}.$$

In the experiments, we control the polarization of the incident wave by rotating half- and quarter-wave plates. In the first case, the polarization of the excitation beam is controlled by the rotation of the half-wave plate rotated by angle *ϕ* between the optical axis of the half-wave plate and the plane of incidence. The polarization plane orientation after a half-wave plate is *Φ* = 2*ϕ*. If the initial laser beam wave has *s*-polarization, the *p*- and *s*- components of the light electric field after the half-wave plate are:

(A6) $$E_{p}=E\sin2\phi =E\sin\Phi ,$$

(A7) $$E_{s}=E\cos2\phi =E\cos\Phi .$$

That is, the incident wave is *p*-polarized at *ϕ* = 45° and *s*-polarized at *ϕ* = 0.

If the polarization of the excitation beam is controlled by the rotation of the quarter-wave plate by φ, at *s*-polarized initial beam the *p*- and *s*-components of the electric field after the wave plate are, respectively:

(A8) $$E_{p}=\frac{1}{\sqrt{2}}E\sin2\varphi ,$$

(A9) $$E_{s}=\frac{1}{\sqrt{2}}E\left(\cos2\varphi +i\right).$$

The incident wave is *s*-polarized at φ=0 and is circularly polarized at φ=±45°.

The amplitude, phase, and polarization of the transmitted wave in the medium are determined by the amplitude and polarization of the incident wave as well as by the angle of the incidence. It is convenient to introduce the wave vector of the transmitted wave $K^{\left(t\right)}=\frac{\omega n}{c}k^{\left(t\right)}$, where $k^{\left(t\right)}=\left\{k_{x}^{\left(t\right)},\;0,\;k_{z}^{\left(t\right)}\right\}$ is the complex vector of the unit length, ${\left|k_{x}^{\left(t\right)}\right|}^{2}+{\left|k_{z}^{\left(t\right)}\right|}^{2}=1$ (see Figure A1).

The wave vector inside the medium can be determined from the conservation of the light wave momentum along the *x*-axis (Snell’s law):

(A10) $$nk_{x}^{\left(t\right)}=k_{x}.$$

That is $k_{x}^{\left(t\right)}=\frac{1}{n}\sin\alpha$. It is convenient to introduce the refraction angle $\sin\alpha_{t}=\frac{1}{n}\sin\alpha$. It is worth noting that in an absorbing medium, $\sin\alpha_{t}$ and $\alpha_{t}$ are complex. The *z*-component of the wave vector of the transmitted wave is $K_{z}^{\left(t\right)}=-\frac{\omega n}{c}\cos\alpha_{t}=-\frac{\omega n}{c}\sqrt{1-\frac{\sin^{2}\alpha }{n^{2}}}$. We can introduce a complex vector of unit length $k^{\left(t\right)},$ describing propagation direction of the light wave in an absorbing medium:

(A11) $$k^{\left(t\right)}=m\left\{\sin\alpha_{t},\;0,-\cos\alpha_{t}\right\}=m\left\{\frac{\sin\alpha }{n},\;0,-\sqrt{1-\frac{\sin^{2}\alpha }{n^{2}}}\right\},$$

where $m={\left({\left|\sin\alpha_{t}\right|}^{2}+\left|\cos\alpha_{t}\right|\right)}^{-1/2}={\left({\left|\frac{\sin\alpha }{n}\right|}^{2}+\left|1-\frac{\sin^{2}\alpha }{n^{2}}\right|\right)}^{-1/2}$.

Unit vector along *s*-polarization of the transmitted wave is the same as that of the incident wave,

(A12) $$s^{\left(t\right)}=s=\frac{\left[k\times N\right]}{\left|\left[k\times N\right]\right|}=\left\{0,-1,\;0\right\},$$

then unit vector along *p*-polarization of the transmitted wave is

(A13) $$p^{\left(t\right)}=\frac{\left[s\times k^{\left(t\right)}\right]}{\left|\left[s\times k^{\left(t\right)}\right]\right|}=\left\{-k_{z}^{\left(t\right)},\;0,\;k_{x}^{\left(t\right)}\right\}.$$

By using textbook expressions for the transmission coefficients of the *s*- and *p*-polarized light wave [60]:

(A14) $$t_{s}=\frac{2\cos\alpha }{\sqrt{n^{2}-\sin^{2}\alpha \;}+\cos\alpha },$$

(A15) $$t_{p}=\frac{2n\cos\alpha }{n^{2}\cos\alpha +\sqrt{n^{2}-\sin^{2}\alpha \;}},$$

one can present the vector of the electric field inside the material in the following form:

(A16) $$E^{\left(t\right)}=st_{s}E_{s}+p^{\left(t\right)}t_{p}E_{p}.$$

The Cartesian components of the electric field can be presented in the following form:

(A17) $$E_{x}^{\left(t\right)}=p_{x}^{\left(t\right)}t_{p}E_{p}=-k_{z}^{\left(t\right)}t_{p}E_{p}=mt_{p}E_{p}\cos\alpha_{t},$$

(A18) $$E_{y}^{\left(t\right)}=t_{s}E_{s},$$

(A19) $$E_{z}^{\left(t\right)}=p_{z}^{\left(t\right)}t_{p}E_{p}=k_{x}^{\left(t\right)}t_{p}E_{p}=mt_{p}E_{p}\sin\alpha_{t}.$$

The Equations (A17)–(A19) describe the electric field inside the material and allow us to express the PDE and SPGE photocurrents.
